# Delineation of molecular interactions of plant growth promoting bacteria induced β-1,3-glucanases and guanosine triphosphate ligand for antifungal response in rice: a molecular dynamics approach

**DOI:** 10.1007/s11033-021-07059-5

**Published:** 2021-12-16

**Authors:** Yachana Jha, Budheswar Dehury, S. P. Jeevan Kumar, Anurag Chaurasia, Udai B. Singh, Manoj Kumar Yadav, U. B. Angadi, Rajiv Ranjan, Minaketan Tripathy, R. B. Subramanian, Sunil Kumar, Jesus Simal-Gandara

**Affiliations:** 1 N. V. Patel College of Pure and Applied Sciences, S.P. University, Anand, 388315 India; 2 ICMR-Regional Medical Research Centre, Bhubaneswar, 751023 India; 3 ICAR-Directorate of Floricultural Research, Pune, 411036 Maharashtra India; 4 ICAR- Indian Institute of Vegetable Research, Varanasi, 221305 India; 5 ICAR-National Bureau of Agriculturally Important Microorganisms, Mau, 275103 Uttar Pradesh India; 6 SRM University, Sonepat, 131029 Haryana India; 7 Dayalbagh Educational Institute, Agra, 282005 Uttar Pradesh India; 8 Adichunchunagiri University, Javarana Hally, 571448 Karnataka India; 9 BRD School of Bioscience, Sardar Patel University, Anand, 388120 Gujarat India; 10 ICAR-Indian Agricultural Statistics Research Institute, New Delhi, 110012 India; 11 Nutrition and Bromatology Group, Department of Analytical and Food Chemistry, Faculty of Science, Universidade de Vigo, E32004 Ourense, Spain

**Keywords:** Biotic stress, Dot blot analysis, β-1,3-glucanases, GTP ligand, Molecular dynamics simulation

## Abstract

**Background:**

The plant growth is influenced by multiple interactions with biotic (microbial) and abiotic components in their surroundings. These microbial interactions have both positive and negative effects on plant. Plant growth promoting bacterial (PGPR) interaction could result in positive growth under normal as well as in stress conditions.

**Methods:**

Here, we have screened two PGPR’s and determined their potential in induction of specific gene in host plant to overcome the adverse effect of biotic stress caused by *Magnaporthe grisea*, a fungal pathogen that cause blast in rice. We demonstrated the glucanase protein mode of action by performing comparative modeling and molecular docking of guanosine triphosphate (GTP) ligand with the protein. Besides, molecular dynamic simulations have been performed to understand the behavior of the glucanase-GTP complex.

**Results:**

The results clearly showed that selected PGPR was better able to induce modification in host plant at morphological, biochemical, physiological and molecular level by activating the expression of β-1,3-glucanases gene in infected host plant. The docking results indicated that Tyr75, Arg256, Gly258, and Ser223 of glucanase formed four crucial hydrogen bonds with the GTP, while, only Val220 found to form hydrophobic contact with ligand.

**Conclusions:**

The PGPR able to induce β-1,3-glucanases gene in host plant upon pathogenic interaction and β-1,3-glucanases form complex with GTP by hydrophilic interaction for induction of defense cascade for acquiring resistance against *Magnaporthe grisea.*

**Graphical abstract:**

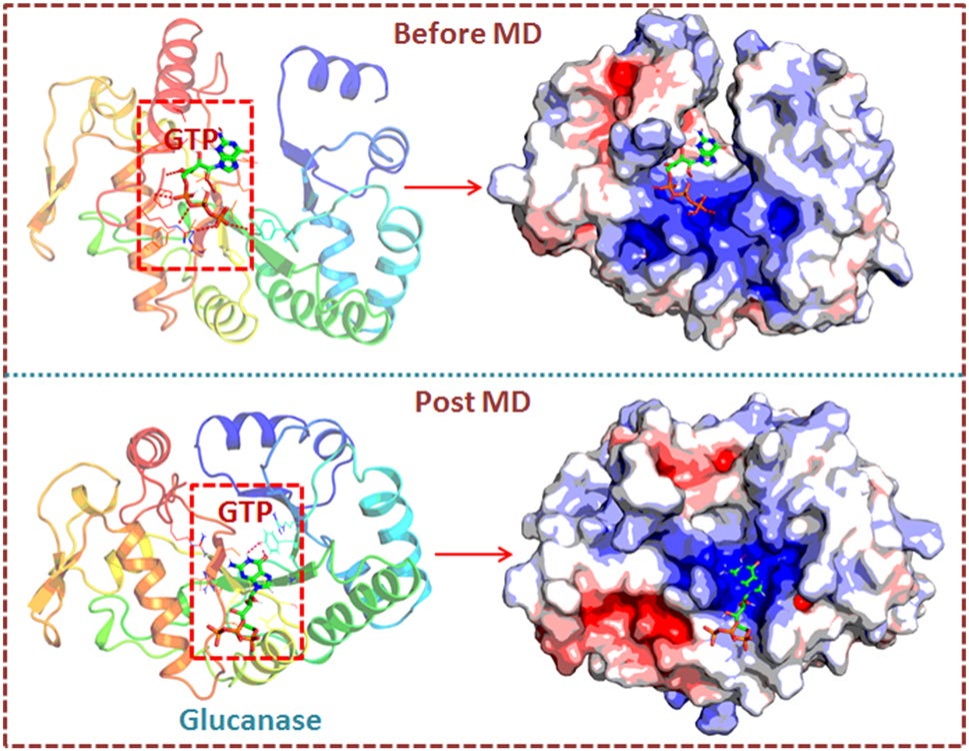

**Supplementary Information:**

The online version contains supplementary material available at 10.1007/s11033-021-07059-5.

## Introduction

Different types of soil microbes continuously interact with roots of plant in search of food, but it does not result in plant disease frequently, which implies that the plants develop an efficient mechanism to discriminate between normal and potential pathogens. Efficient defense mechanism is induced in plants to check the colonization of root by surrounding phytopathogens [1]. Upon sensing pathogens in their vicinity, a series of defense activities are activated in plant, like stimulation of antimicrobial compounds, closure of stomata, accumulation of lytic enzymes, cell wall lignification, rapid oxidative burst and production of multi-level defense signals that work in specific coordination to block the pathogens interaction with the plant cell due to which plant occasionally get infected by pathogens [2–4].

Plants respond to the pathogens by varying degrees of defense responses. During plant infection, the penetration of pathogens usually rely on the precise time-course of the pathogen discernment by the host plant cells and initiate various biochemical reaction resulting in production of reactive oxygen species (ROS), secondary metabolites, and pathogenesis-related proteins (PRs) and all employed in combination to activate proper defense mechanism against the pathogen infection [5–7]. Synthesis of pathogenesis-related (PR’) proteins like β-1,3 glucanases, chitinases, peroxidases, hydroxyl proline-rich glycoproteins, phenylpropanoid and flavonoid directly or indirectly play an important role in the process of pathogenesis. These β-1,3-glucanases (EC 3.2.1.39) present in plants play key role in trafficking of materials across the cell membrane, cell division as well as in defense against pathogen. β-1,3-glucanases play important roles in fighting against the pathogenic fungus by PR-2 family proteins in plants [8, 9]. β-1,3-glucanases catalyze the breakdown of 1,3-D-glucosidic linkages in β-1,3-D-glucan, which is the main cell wall component in fungi.

The plant roots remain intensely colonized by different types of soil microorganisms like bacteria and fungi, as root exudates represent a rich source of nutrients for their growth and establishment. This group of bacteria is also known as plant growth promoting rhizobacteria (PGPR), which play an effective role in plant growth promotion as well as in alleviating the adverse effects of various biotic and abiotic stresses and are involved in many metabolic and physiological processes of plant [10]. Such microorganisms form an association with the plant, and in turn protect the host plant from adverse environmental stresses by producing or regulating the bioactive compounds such as β-1,3-glucanases (lytic enzymes) and antibiotics.

These microbial bioactive compounds have imperative responsibilities in the plant development and growth by participating in various mechanisms like providing resistance against biotic and abiotic stress factors, protecting plants from numerous phytopathogens and improving the nutrient acquisition ability, and inducing the plant development and yield under stress conditions [11]. The PGPR are non-pathogenic soil bacteria, which could be employed as a sustainable alternative to manage and control the phytopathogens than harmful chemical pesticides.

The PGPR also produce wide range of PR proteins, which coordinate in developmental control and induce defense response against pathogens in the host plant. Interaction of PGPR with the host plant result in induction of induced systemic resistance (ISR). The PGPR mediated ISR are different from pathogen-induced systemic acquired resistance (SAR) in many aspects at molecular level. While pathogen induced SAR is similar to PGPR mediated ISR in developing resistance towards a broad variety of phytopathogens and spread the resistance to uninfected plant parts [12]. The aim of the present study was to explore the potential of PGPR in the induction of β-1,3- glucanase gene expression after fungal interaction in paddy crop. Besides, molecular modeling of glucanase and its interaction study with GTP ligand in silico was performed.

The interactions of glucanase with GTP can be explored through the molecular dynamics simulation (MD) studies. Therefore, in the present study we employed the molecular modeling technique along with MD simulations to infer the probable mode of binding of glucanase to GTP. The principal component analysis (PCA), ensemble clustering and molecular mechanics-based binding free energy (MM/PBSA) calculations were employed to elucidate the global motions and the energetic components which drive the interaction of glucanase with GTP. To the best of our knowledge, this is the first such report on fungal pathogen infected paddy crop, where in-silico study of β-1,3-glucanases interaction with GTP ligand has been complemented with the wet lab experiments.

## Materials and methods

### Isolation, identification and inoculation of PGPR

In this study, two strains of *Pseudomonas pseudoalcaligenes* and *Bacillus pumilus* were isolated from the paddy rhizosphere as per the procedure illustrated by Jha et al. [13]. Among them, the isolated strains were identified by PCR amplification of 16S rDNA using 16S universal primer 8F: 5′AGAG TTTGATCCTGGCTCAG3′ and 1510R: 5′GGCTACCTTG TTACGTA3′. The amplified products were sequenced, analyzed by BLAST and submitted to the NCBI database with accession numbers EU921258 and EU921259, respectively. The seeds of rice variety GJ-17 were procured from main rice research center, Navagam, Anand, Gujarat. Aseptic rice seedlings (four days age) of both single and mixture were transferred to culture vials having Hoagland’s nutrient medium (400 µL) coupled with 400 µL of micronutrients and agar 1% (w/v) in 40 mL distilled water. Before transfer, *B. pumilus* and *P. pseudoalcaligenes* bacteria were added to the medium (6 × 10^8^ cfu mL^−1^) and incubated at 27 °C in a 12 h light–dark cycle in a growth chamber.

### RNA extraction and RT-PCR from isolated RNA

One gram of fresh paddy leaves from all the treatments (eight samples –four controls, plant inoculated with *Bacillus* sp, plant inoculated with *Pseudomonas* sp. and plant inoculated with both in absence of pathogen similarly four others in presence of pathogen) were used for the extraction of RNA. Extraction of RNA was performed in complete nuclease free condition using diethyl pyrocarbonate (DEPC) as per the protocol [14]. The obtained RNA was quantified by nano-drop spectrophotometer, determined quality and stored at − 20 °C for further use.

RT-PCR of the extracted RNA was performed with the help of RT- PCR Kit (Bangalore Genei) instructions. Two degenerate primers for β-1,3-glucanase with random oligo dT primers were used for the amplification of β-1,3-glucanase genes. The gene specific primers reverse 5′-GGTTCTCGTTGAACATGGCGA-3′and forward 5′-GTGTCTGCTATGGCGTTGTCG-3′; were designed and synthesized by Invitrogen Ltd. The reactions were carried out in duplicate for the different primer combinations. PCR amplification of cDNA was carried out in the next step of RT-PCR reaction with 2 µL each of forward and reverse primers, 1.25 µL of 10× assay buffer, 1 µL dNTP mix, 0.25 µL *Taq* DNA polymerase, 3 µL cDNA and 3µL nuclease free water. The PCR conditions were: initial denaturation at 94 °C for 2 min, denaturation 94 °C for 1 min, annealing of primer for 1 min, extension at 72 °C for 4 min and 72 °C for 10 min for 30 cycles.

### DNA elution from the gel

DNA band of interest was excised from the agarose gel using a sharp sterile blade. Gel piece was weighed and larger piece was crushed into smaller pieces. 2.5 mL of sodium iodide solution was added and the gel was incubated in a water bath at 50–55 °C for 2 to 3 min to solubilize it. To the solubilized gel, 15 µL of glass solution was added to the sample containing 5 µL of DNA. The contents were mixed thoroughly and spun at 12,000 g for 30 s. Supernatant was discarded and the pellet containing DNA was taken and wash buffer was added to remove the glass solution, vortexed and spun at 12,000 g for 30 s and again the supernatant was discarded. The tubes were incubated at 37 °C in the water bath for 10 min. For elution of DNA, the pellet was re-suspended in 1X TE buffer, vortexed and incubated at 45 °C for 5 min. This step was repeated thrice, all the fractions were pooled and finally centrifuged at 5000 g for 2 min to remove any traces of glass solution. Efficiency of elution was checked on 1.5% (w/v) agarose gel. The eluted gel band was sequenced by Xplorigen Technologies and obtained sequence was analyzed for multiple sequence alignment by Clustal W software. The ExPASy translate tool was used to translate the obtained nucleotide sequences into amino acid sequences. The longest open reading frame was selected for protein BLAST search against non-redundant database of NCBI to search for similar proteins.

### RNA dot blot assay

Fluorescence-labeled probes for RNA dot blot assay were designed in the lab and obtained from Genei, Bangalore, India. The purified 6 µg of RNA was directly applied on the nylon membrane by automatic dispenser for RNA dot blot assay and was vacuum baked for 2 h at 80 °C for fixation of RNA on the membrane. The membrane was treated with pre-hybridization buffer to block the free sites on the membrane to prevent unspecific binding of probe. Then biotin labeled gene specific probe was used for hybridization at optimum temperature and salt concentrations. The hybridized membrane was washed 2–3 times to remove unbounded probe and used for detection. Diluted HRP- streptavidin conjugate was used for detection at room temperature in the presence of substrate solution with gentle swirling until a blue colour band developed. The detection reaction was according to the manufacturer’s instruction (Bangalore Genei). The spot density of the RNA dot blot was measured by Alpha Ease FC Imaging software (Alpha Innotech).

### Molecular modeling of glucanase and docking studies with GTP

The amino acid sequence of beta-1,3-glucanase (*Oryza sativa* indica) was extracted from NCBI database (*O. sativa*; accession no. ADO34999). Templates for the comparative modeling were identified using the BLASTp search against the Brook Haven Protein Data Bank (PDB) with default parameters [15–18]. The sequence alignments of the target and template were carried out using Multalin program [19]. The 3-dimensional model of beta-1,3-glucanase was generated using the program MODELLER version 9.21 [20, 21].

Roughly 200 models were generated by the modeller based on the lowest value of the probability density function and the fewest restraint violations. Out of the generated models were used for further downstream analysis. The energy minimization was performed on the selected model. We first conducted energy minimization with side chains and then subsequently applied it to the main chain of a Cα backbone. All the calculations were performed using ACCELRYS Discovery Studio 2.5 (Accelrys Inc. San Diego, CA 92,121, USA) software suites. STRIDE was used for the prediction of secondary structure of the modelled glucanase protein. The modelled structure was then superimposed on the template without altering the coordinate systems of atomic position in the template. The residue profiles of the three-dimensional models were further checked using VERIFY3D (http://www.doe-mbi.ucla.edu/Services/Verify3D.html). PROCHECK analysis was performed to assess the stereo chemical properties of the three-dimensional models and Ramachandran plots [22].

The homology modelled structure of glucanase was used for docking with the ligand GTP. For docking analysis, we downloaded the structure of GTP from the PubChem database (http://pubchem.ncbi.nlm.nih.gov). An attempt was made to understand the binding of GTP on glucanase interface AutoDock version 4.2 [23] and AutoDocktools 1.5.6.were applied. Prior to this, protein were prepared and added polar-hydrogen atoms using Gasteiger charges calculation. Ligand centered map was developed having 0.375 Å spacing with grid dimensions of 46 × 46 × 46 Å^3^ (x-y-z) encompassing the binding interfaces of the protein. While performing docking, default settings were applied with GA run to 300. The best docked conformation was selected by MD optimization considering the parameters such as higher hydrogen-bonds, least free energy of binding and interatomic-bonding patterns. BIOVIA Discovery Studio Visualizer version 4.5 and PyMOL were deployed to elucidate the inter-molecular contacts between GTP with glucanase.

### Molecular dynamics simulation of glucanase-GTP complex

MD simulations of glucanase-GTP complex were done to explore the stability, conformational flexibility and dynamic behavior of all-atoms [24, 25]. Ligand topology was deduced from CHARMM general force field (https://cgenff.umaryland.edu/) [26] and force fields of protein building was done using GROMACSv2019.4 package [27]. The structure was solvated in a cubic water box in TIP3P water model. The solvated system was then electro-neutralized by addition of 0.15 M NaCl. Energy minimization was performed to remove bad contacts in the complex system using a steepest descent algorithm in 5000 steps. Equilibration was conducted through NVT and NPT ensembles for 10 ns at 300 K. The non-hydrogen atoms of the ligand were restrained. Using leapfrog integrator, production of MD was carried out for 50 ns. GROMACS utility toolkits were employed to understand the structural dynamics and intrinsic stability of the Glucanase-complex system. Using the snapshots from MD trajectory, a number of dynamics stability parameters were computed to understand the structural-dynamics of the MD system which includes root mean square deviation (RMSD), Cα-root mean squared fluctuations (RMSF) and intermolecular hydrogen bond (H-bond) distributions. XMGrace was employed to generate the 2D graphs and images were rendered using BIOVIA DSV and PyMOL.

## Results

As PGPR’s are supposed to develop resistance against pathogen, which remain coupled with the corresponding induction in plant gene expression. Total RNA was isolated from the PGPR inoculated paddy leaves after one week of fungal infection and used for the synthesis of cDNA by reverse transcriptase enzyme. The obtained product was used for subsequent amplification of β-1,3-glucanases gene by PCR using the gene specific primers (Fig. S1). Induction of β-1,3 glucanase gene in PGPR inoculated plant after pathogen infection was characterized by RT-PCR analysis. The obtained sequences were gathered to establish a consensus sequence. It was uniformly inducted by the inoculation of paddy with either single isolate or in combination. The β-1,3-glucanases gene length of 1.05 kb DNA was assembled and submitted to NCBI data base having accession no HM569719.1.

### RNA dot blot assay

The differential changes in mRNA level of PGPR inoculated plant after specific stress treatment was analyzed by RNA-dot blot assay. The differential expression of β-1,3-glucanases gene at the level of transcription under each set of condition was confirmed by RNA dot blot using a eukaryotic translation factor eTEF1α as an internal standard. The spot density for β-1,3glucanase showed remarkable variation in RNA dot blot assay as observed under Alpha Ease FC Imaging software (Alpha Innotech). The control plant showed no spot in RNA dot blot assay, but PGPR inoculated plants showed remarkable differences with and without fungal pathogen infection (Fig. S2 and Table S1). The highest RNA dot blot spot density was observed in plants co-inoculated with *P. pseudoalcaligenes* followed by plants inoculated with both the PGPR in presence of pathogen. The spot was also observed in the plants only inoculated with PGPR in absence of pathogen. Its sequence (Table S2) homology was analyzed by ClustalW software in which it showed close homologies with *Oryza sativa* mRNA complete cds (AF337174.1), *Oryza sativa* japonica group cDNA clone (AK069096.1) and *Oryza sativa* (indica cultivar-group) cDNA clone (CT837570.1) with endo-1,3-beta-glucanase.

### Molecular modeling of glucanase and docking studies

The 3D structure of the beta-1,3-glucanase(*Oryza sativa* indica) has not been solved yet experimentally. Thus, we built a 3D model of the beta-1,3-glucanase protein using homology modelling approach. BLASTp search against PDB resulted in four templates i.e., 1GHS_A:*Hordeum vulgare*; 2CYG_A: *Musa acuminate;* 4HPG_A: *Hevea brasiliensis;* 3UR7_A: *Solanum tuberosum* **(**Table S3**)**. Based on the pair-wise sequence alignment between glucanase and the templates using Multalin program protein models were predicted (Fig. S3). After obtaining the final model, overall stereo-chemical qualities of the model were evaluated to ensure its suitability for carrying out the further studies.

Stereo chemical quality of modelled glucanase structure (Fig. S4A, B) was explored using Ramachandran plot. Procheck was employed to validate the rationality of homology model. Ramachandran plot analysis displayed the dihedral angles Φ against Ψ of amino acid residues in the predicted model (Fig. S4C), where 93.1% residues were found to be in the most favored region and none of the residues with bad geometry (outlier region of Φ and Ψ plot) depicting good quality of the model. ERRAT program was used to assess the overall quality factor of the modelled protein by determining the false statistics of bad non-bonded interactions within the structure.

The overall quality factor of the modelled structure was 86% which indicates that the proposed model is of good quality. Additional analysis of the structure using the Profile-3D score showed comparatively a good score of 98.45% indicating the accuracy of homology model. Analysis of the predicted model using ProQ server indicated LG-score of “extremely good model” and Max-Sub of “very good model” quality measures. ProSA-Web (Fig.S4D) analysis exhibited the energy profile and Z-score of the model. Z-score for the predicted model was found to be within the range of scores typically found for the native proteins of similar size while the plot of energies of each residue revealed that entire calculated values were negative. Overall, the various model validation servers suggested that the generated model is reliable (Table 1) and thus can be used for docking studies with higher confidence. Furthermore, we made structural imposition of our proposed model with structural templates (1GHS: *Hordeum vulgare*; 2CYG:*Musa acuminata*; 4HPG:*Hevea brasiliensis*; 3UR7: *Solanum tuberosum*) displayed least RMSD (below 0.8 Å) indicating the overall good quality of our model structure (Fig. S5).

**Table 1:** Model validation statistics of glucanase subunit of *rice* using various structural evaluation servers

Model validation servers	Model quality parameters	Validation scores
Procheck (Ramachandran plot)	Most favored regions (%)	93.1
	Additional allowed regions (%)	6.4
	Generously allowed regions (%)	0.5
	Disallowed regions (%)	0.0
Verify 3D	Averaged 3D-1D score ≥ 0.2(%)	98.45
ERRAT	Overall quality (%)	86.0
ProSA	Z score	− 8.42
ProQ	LG score	6.33
	Max Sub	0.61
Prove	Z score mean	− 0.06
METAMQAP-II	GDT_TS	58.69

**Fig. 1:**
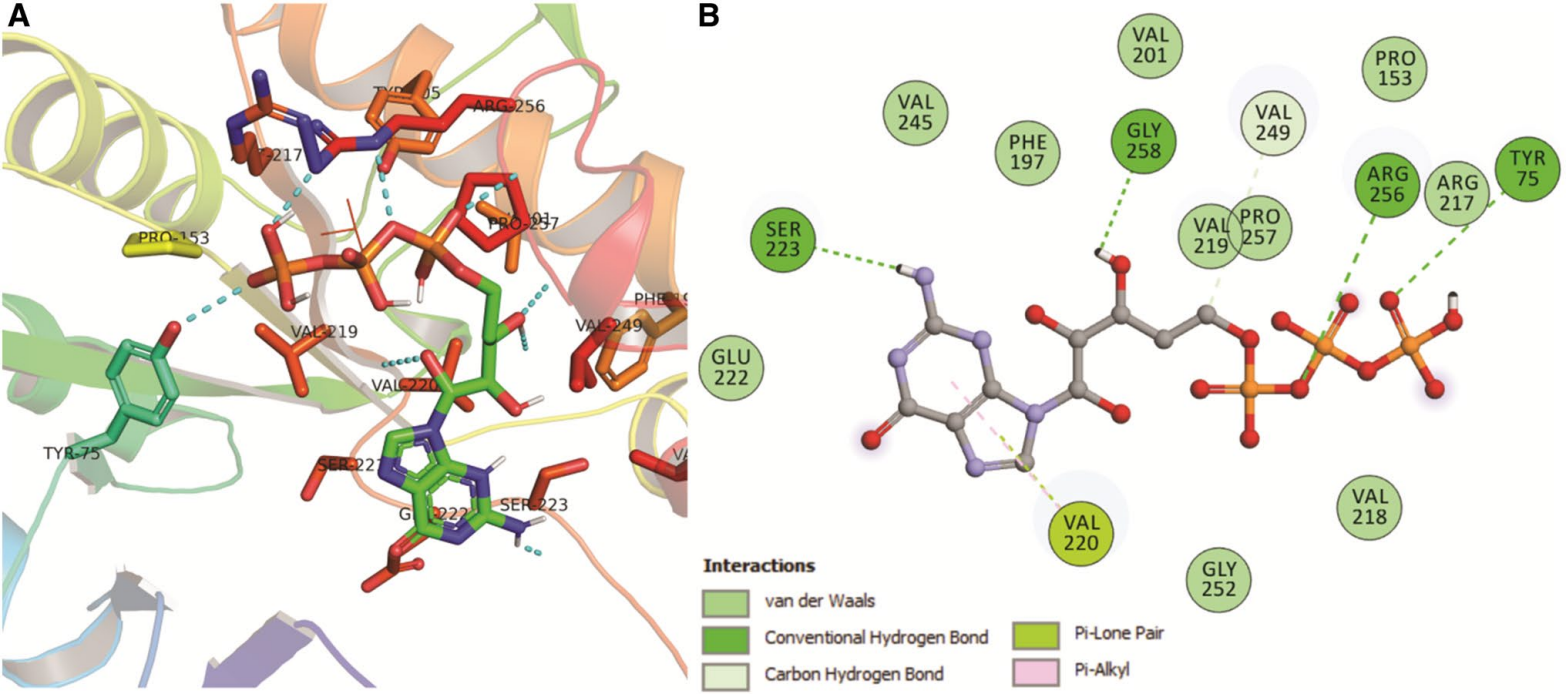
Inter-molecular contact analysis of GTP with modelled glucanase obtaining after docking using AutoDock. **A** Interaction of GTP with modelled glucanase using PyMOL where the dotted lines depict hydrogen bonds and the interacting amino acids along with the ligand (stick format). **B** Non-bonded contact of GTP with glucanase using BIOVIA DSV (hydrogen bond forming amino acids marked in dark green). (Color figure online)

Molecular docking was performed using AutoDock to understand the mode of interaction of GTP in the ligand binding interface of glucanase. As evidenced from the top ranked conformation (as shown in Fig. 1A, B), the GTP prefers to bind in the cavity formed in glucanase receptor (Table 2). The resulted docking conformation from molecular docking experiment having lowest binding energy of − 4.97 kcal/mol was selected as the representative structure for protein-ligand interaction studies. Tyr75, Arg256, Gly258, and Ser223 of glucanase formed four crucial hydrogen bonds with the GTP at an atomic distance 3.18, 3.03, 2.24, and 2.03 Å, respectively. While the Val220 of glucanase displayed hydrophobic contacts with GTP (Fig. 1).

**Table 2:** Intermolecular contacts of the docked conformation of Glucanase-GTP complex obtained from AutoDock analyzed using BIOVIA DSV

Interacting pairs	Distance	Type	Category
TYR75: OH: GTP: O17	3.17837	Hydrogen bond	Conventional hydrogen bond
ARG256:NE: GTP: O8	3.03002	Hydrogen bond	Conventional hydrogen bond
GTP:H40: GLY258: OXT	2.24216	Hydrogen bond	Conventional hydrogen bond
GTP:H44: SER223:O	2.02791	Hydrogen bond	Conventional hydrogen bond
GTP:C27: VAL249:O	3.48525	Hydrogen bond	Carbon hydrogen bond
VAL220:O: GTP	2.77519	Other	Pi-lone pair
GTP: VAL220	4.62512	Hydrophobic	Pi-alkyl
GTP: VAL220	5.02469	Hydrophobic	Pi-alkyl

### MD trajectory analysis

**Fig. 2:**
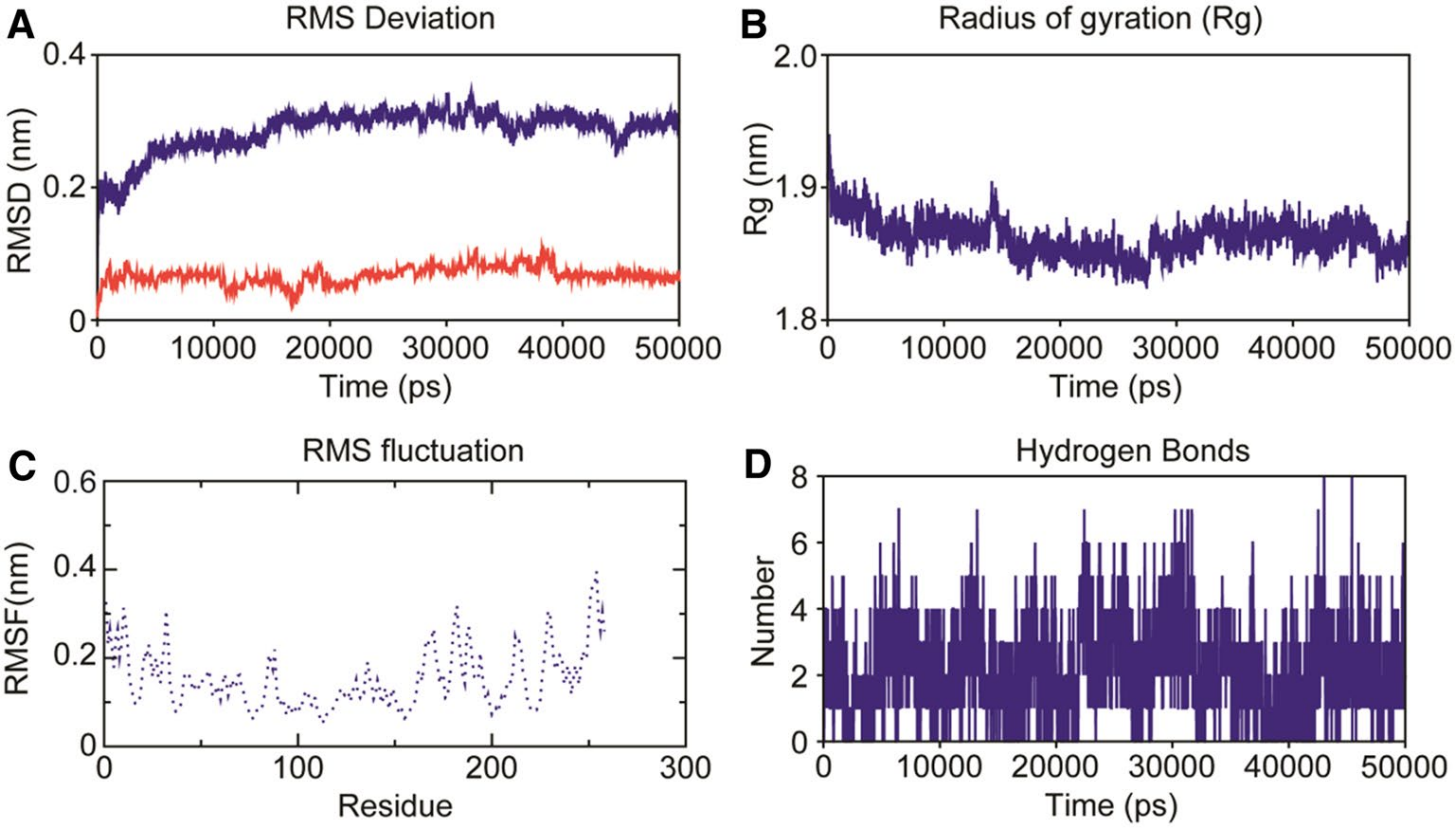
Dynamics stability of the glucanase-GTP complex system during 50 ns MD. **A** Backbone RMSD of the complex and the ligand over the time scale of 50 ns. **B** Radius of gyration profile indicating the compactness of the system during 50 ns. **C** The C-α RMSF profile of the glucanase-GTP complex during the last 30 ns MD. **D** Intermolecular H-bond analysis of GTP with glucanase during 50 ns MD

**Fig. 3:**
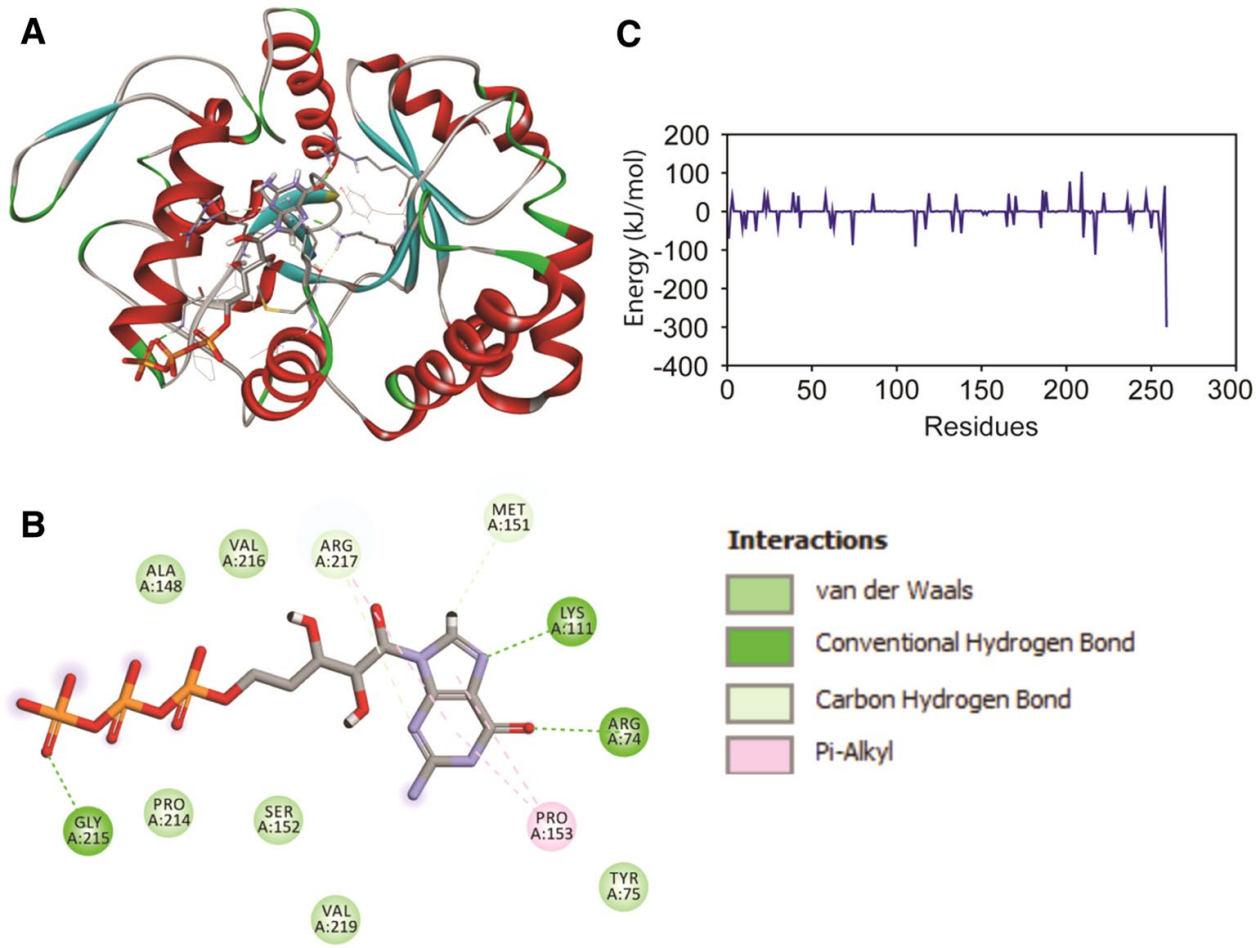
Inter-molecular contact analysis of the glucanase-GTP complex post MD and the binding free energy decomposition analysis displaying the contribution each residue towards binding free energy. **A** Snapshot of the Glucanase-GTP complex obtained post MD using clustering approach. **B** Inter-molecular contacts of the top ranked cluster displaying the non-bonded contacts. **C** Free energy decomposition analysis displaying the energetic contribution of each residue towards binding free energy

To understand the dynamic behavior of glucanase-GTP complex, we performed all-atoms MD simulations of complex using GROMACS. To gauge the stability and conformational flexibility of the complex, we computed various properties of the system. The backbone RMSD analysis offers vital information on the stability of protein-ligand complexes and the time when simulation reached equilibrium. RMSD profiles of glucanase-GTP complex displayed least deviation with average RMSD of ~ 0.32nm during the simulation period and by the time of 20 ns system achieved convergence (Fig. 2A). In addition, the RMSD of ligand (as shown in red line in Fig. 3A) was also found to be stable (below 0.1 nm) with minimum deviation. In toto, the docked confirmation remained accurate by manifesting least backbone deviation and stable for 50 ns timescale.

Radii of gyration determines the robustness of the system; where, gyradius of ~1.85nm implies the consistency of size and shape of the system while performing simulation (Fig. 2B).Glucanase-GTP complex flexibility has been evaluated suing Cα RMSF analysis (Fig. 2C). As compared to the N-terminal end, the C-terminal end had major fluctuations with higher RMSF values, while the loops also displayed higher peaks indicating flexible nature of the mobile loops during MD.

The intermolecular hydrogen bonds (H-bonds) in a protein-ligand complex plays dynamic role in stability and molecular recognition process. The intermolecular H-bonds were calculated with respect to time during the 50 ns MD simulation to see the dynamic stability of glucanase-GTP complex (Fig. 2D). We observed an average of ~ 4.04 H-bonds/time frame during the 50 ns time scale of MD. Snapshots from MD revealed that some of the H-bonds were broken during MD simulation, however, later they well remunerated by new non-bonded contacts. Minute observation of the ligand with respect to the initial starting structure used MD revealed that the GTP prefers to reorient within the ligand binding site during MD but form close tight network of hydrogen bonds and non-bonded contact with glucanase of rice. Clustering analysis through RMSD-based clustering employed in *gmx cluster* exposed the crucial residues of glucanase involved in the important interaction with GTP (Table S4). Arg74, Lys111 and Gly215 formed strong H-bonds while, Pro153and Arg217 formed hydrophobic pi-alkyl contacts with the ligand GTP (Fig. 3A, B).

The results from PCA have been summarized in Fig. S6. Porcupine plots of top two PCs displayed prominent inward motion in the encircled helical segments of Glucanase. Mostly the distal end at the terminals displayed high degree of in-ward motion. We also computed binding free energy of the glucanase-GTP complexes using snapshots from MD trajectory through MM/PBSA approach implemented in *g_mmpbsa* tool. A total of 200 snapshots from the last 20 ns of MD were used for computing the binding free energy. Among the energetic terms Van der waals energy, electrostatic energy and SASA energy contributed to the total binding free energy of the system while polar solvation showed positive energy i.e., opposes the binding (Table 3).

**Table 3:** Binding free energy of the Glucanase-GTP complex estimated using MM/PBSA approach

Energetic terms	Energetic contribution (kJ mol^−1^)
van der Waal energy	− 109.27 ±1.10
Electrostatic energy	− 748.29 ± 9.85
Polar solvation energy	290.56 ± 7.79
SASA energy	− 12.95 ± 0.17
Binding energy	− 579.78 ±5.42

To find out the contribution made by each residue towards negative free energy, we also computed the free energy decomposition analysis which revealed a number of amino acids from the binding pocket contributes significantly towards the total binding free energy (Fig. 3C). The contribution made from electrostatic energy signifies the glucanase-GTP complex possess a number of electrostatic contacts as evident from the non-bonded contact analysis. We also extracted the top ranked cluster from MD trajectory to inspect the inter-molecular contacts mediated by glucanase with GTP (table which perfectly fits with the energetic contribution derived MM/PBSA analysis.

## Discussion

Plant–microbe interactions are a common and natural event, which has either beneficial or hostile effect on plant host. Plant also continuously encounter with a wide range of pathogenic microbes having deleterious effect on host plant [28]. A plethora of physical and chemical defense systems have been developed by the plants during the evolutionary process to block the hostile interactions with the different biotic factors. So plants develop a multilayer complex networking pathways to fight with the pathogen, which include the synthesis of PR new proteins, cell lignifications, phytohormone stimulation and induction of defense related gene. Among these chemical defense systems, inhibition of biochemical and enzymatic activities of pathogens or accumulation of lytic secondary metabolites to inhibit the growth or kill the pathogens are predominant mechanisms.

Intensive interactions of microbes with plant roots always take place- as it is a source of diverse and rich microbial foods due to which it acts as active ecological niche [29]. PGPR used to adapt to the specific plant environment in which they colonize and therefore have the higher metabolic potential. Such interaction of plant with PGPR helps in maintaining the plant health by improving the nutrient status, resistant against phyto-pathogens and also to the environmental stresses. The PR protein,β-1,3 glucanases produced by PGPR in host plant are responsible for hydrolyzing the cell walls of fungus and releasing of β-1,3-glucan, so directly involved in the protection of plant against the wide range of fungal pathogens [30]. The released β-1,3-glucan like oligosaccharides can act as an elicitor, which elicit plant defense mechanism. The plant surveillance systems are able to recognize these elicitors, and induce the signaling cascades for the activation of systemic and localized defense responses against the wide range of phytopathogens.β-1,3-glucanases is highly effective against fungal pathogen attack as it degrades the fungal cell wall during the pathogen attack.

In this study, we observed that inoculation of paddy with *P. pseudoalcaligenes* and *B. pumilus* enhanced the plant protection against fungus *Magnaportha grisea*. There are several reports mentioning that inoculation of plants with the selective PGPR is able to induce protection against a wide range of pathogens. ISR is triggered by PGPR, which results in activation of phytohormones mediated signaling pathways [31], production of defense modulation proteins and induction of pathogenesis-related genes to develop the plant ability to fight against wide range of pathogens attack. Microbes interaction triggers PGPR signaling compounds like cell wall components, chitin oligomers and lipopolysaccharides that elicit ISR in plant [32]. ISR-inducing PGPR have been shown to be effective for the disease control in tomato [12].

A 3D model of the beta-1,3-glucanase (glucanase) protein using homology modelling approach was constructed after selecting the best template that was validated by several servers. Overall, the various model validation servers suggested that the generated model is reliable and thus can be used for docking studies with higher confidence. Molecular docking was performed to understand the mode of interaction of GTP in the ligand binding interface of glucanase. As evidenced from the top ranked conformation, the GTP prefers to bind in the cavity formed in glucanase receptor. A number of hydrogen bonds mediated by Tyr75, Arg256, Gly258, and Ser223 form strong interaction with GTP while Val220 alone formed hydrophobic contact. To understand the dynamic behavior of glucanase-GTP complex, we performed all-atoms MD simulations of complex using GROMACS. To gauge the stability and conformational flexibility of the complex, we computed various properties of the system. Overall, the complex system displayed the least backbone deviation, indicating that docked conformation is accurate and remained stable over the 50 ns timescale.

Radius of gyration assesses the compactness of the system, where a compact gyradius of ~ 1.85nm for the complex indicates the consistent shape and size of the system during simulation. The residue flexibility of glucanase-GTP complex was examined by performing Cα RMSF analysis. As compared to the N-terminal end, the C-terminal end had major fluctuation with higher RMSF values. The intermolecular hydrogen bonds (H-bonds) in a protein-ligand complex plays dynamic role in stability and molecular recognition process. The intermolecular H-bonds were calculated with respect to time during the 50 ns MD simulation to see the dynamic stability of glucanase-GTP complex. We observed an average of ~ 4.04 H-bonds/time frame during the 50 ns time scale of MD. The residues which formed close contact with the ligand contributed significantly to the overall binding free energy of the complex. Overall, the stability of the complex system and intrinsic dynamics inferred from this study enables us to understand the molecular recognition of GTP with glucanase and advocates to undertake site directed mutagenesis study to confirm the role of ligand binding pocket residues in binding to GTP.

Globally the researchers have been recommending the application of PGPR as plant growth promoting and biocontrol agents, leading to a green and safe sustainable agriculture practices by reducing the various chemical inputs [33, 34]. Usually, applications of compatible microbial consortiums have proven to be better than single inoculant [35]. There are many publications in literature explaining the mechanism behind these actions [31], but none of them have ever proved this phenomenon complementing the in-silico and wet lab experiments at the molecular level. Our investigations will fill this gap in knowledge existing in the literature and will work as a backbone for the future bioinformatics-based investigation of mechanism of PGPR action.

This study showed that association of PGPR with plant roots alone is responsible for the induction of plant defense gene expression. Findings of the study indicate that the mixture of both *P. pseudoalcaligenes*and *B. pumilus* confer better effective resistance by efficient induction of the defense in plant with comparison to the individual isolate. RT-PCR and RNA dot blot analysis both implies that plant defense gene β-1,3-glucanases is induced at significant level in the plant upon inoculation with PGPR to induce the plant defense. To assess the differential level of mRNA production due to induction by PGPR and after fungal infection in plants, RNA-Dot blot assay was used and results showed that PGPR were able to induce significant level of mRNA in plant to provide the defense against fungal pathogen. However, fungal infection does not show any remarkable variation in spot density of RNA in RNA-Dot blot assay.

So, the finding showed that plant inoculated with mixture of *P. pseudoalcaligene and B. pumilus* confers significant resistance against plant pathogen by inducing changes in the associated gene expression in plant. Moreover, such induction in defense associated gene expression in plant is initiated alone by PGPR prior to the pathogenic interaction. The interaction of plant growth promoting bacteria with plant during the initial stage of development induce multi level change in gene expression cause related change in plant physiology to overcome the adverse effect of stresses has been better analyzed by multi-omics to study the modulatation of cellular mechanisms in plants has been also reported by Meena et al. [36].

## Supplementary Information

Below is the link to the electronic supplementary material.

Supplementary material 1 (DOCX 2186.6 kb)
