# Supplementary material for: Delineation of molecular interactions of plant growth promoting bacteria induced β-1,3-glucanases and guanosine triphosphate ligand for antifungal response in rice: a molecular dynamics approach
Source: Mol Biol Rep. 2021 Dec 16;49(4):2579–89. doi: 10.1007/s11033-021-07059-5 (PMC8924079; doi:10.1007/s11033-021-07059-5)
Supplement: Supplementary file 1 — Supplementary material 1 (DOCX 2186.6 kb) [file 11033_2021_7059_MOESM1_ESM.docx]

**Supplementary File**

**Tables**

Table S1. RNA dot blot analysis of β 1,3 – glucanase for spot density

| S.No. | Spot No. | Spot identity  (Total RNA of sample) | IDV | % | Area | AVG |
| --- | --- | --- | --- | --- | --- | --- |
| 1. | 1n | Control | nil | nil | Nil | nil |
| 2. | 2n | Control +*B. pumilus* | 59333 | 10.8 | 522 | 114 |
| 3. | 3n | Control +*P. pseudoalcaligenes* | 54688 | 10.1 | 444 | 123 |
| 4. | 4n | Control + *B. pumilus* + *P. pseudoalcaligenes* | 40056 | 7.3 | 338 | 119 |
| 5. | 5n | Control + pathogen | nil | nil | Nil | nil |
| 6. | 6n | Control +*B. pumilus* + Pathogen | 39120 | 7.1 | 308 | 127 |
| 7. | 7n | Control + *P. pseudoalcaligenes* + Pathogen | 30252 | 5.3 | 222 | 136 |
| 8. | 8n | Control + *B. pumilus* +*P. pseudoalcaligenes* + Pathogen | 19713 | 3.6 | 152 | 130 |
| 9. | 9n | eTEF1α | 49176 | 9.0 | 444 | 111 |
| 10. | 10n | eTEF1α | 41001 | 7.5 | 370 | 110 |
| 11. | 11n | eTEF1α | 31061 | 5.5 | 280 | 110 |
| 12. | 12n | eTEF1α | 34117 | 6.4 | 306 | 111 |

Table S2. Sequence statistics for β-1,3 glucanase cDNA gene

| DNA molecule | Glucanase Assembled |
| --- | --- |
| Length | 774 base pairs |
| Molecular Weight (single stranded) | 237396.00 Daltons |
| Molecular Weight (double stranded) | 472727.00 Daltons |
| G+C content | 66.02% |
| A+T content | 32.95% |

| Nucleotide | Number | Mol% |
| --- | --- | --- |
| A | 144 | 18.60 |
| G | 271 | 35.01 |
| C | 240 | 31.01 |
| T | 111 | 14.34 |
| N | 8 | 1.03 |

Table S3. Templates selected for homology modeling of glucanase of rice

| PDB ID | Source | Query coverage | E-value | % Identity |
| --- | --- | --- | --- | --- |
| 1GHS_A | *Hordeum vulgare* | 99% | 5e-105 | 62.11 |
| 2CYG_A | *Musa acuminata* | 99% | 2e-85 | 57.69 |
| 4HPG_A | *Hevea brasiliensis* | 99% | 3e-82 | 49.81 |
| 3UR7_A | *Solanum tuberosum* | 99% | 2e-71 | 45.83 |

Table S4. Intermolecular contacts of Glucanase-GTP complex after MD (top ranked cluster from clustering analysis was used for inter-molecular contact analysis)

| Interacting Pairs | Distance (Å) | Type | Category of bonding |
| --- | --- | --- | --- |
| A:ARG74:HH21 - B:GTP:O11 | 2.05 | Hydrogen Bond | Conventional Hydrogen Bond |
| A:LYS111:HZ3 - B:GTP:N20 | 1.91 | Hydrogen Bond | Conventional Hydrogen Bond |
| A:GLY215:H - B:GTP:O17 | 1.67 | Hydrogen Bond | Conventional Hydrogen Bond |
| A:ARG217:CD - B:GTP:N19 | 3.35 | Hydrogen Bond | Carbon Hydrogen Bond |
| B:GTP:H29 - A:MET151:O | 2.94 | Hydrogen Bond | Carbon Hydrogen Bond |
| B:GTP - A:PRO153 | 4.64 | Hydrophobic | Pi-Alkyl |
| B:GTP - A:PRO153 | 4.18 | Hydrophobic | Pi-Alkyl |
| B:GTP - A:ARG217 | 5.13 | Hydrophobic | Pi-Alkyl |

**Figures**


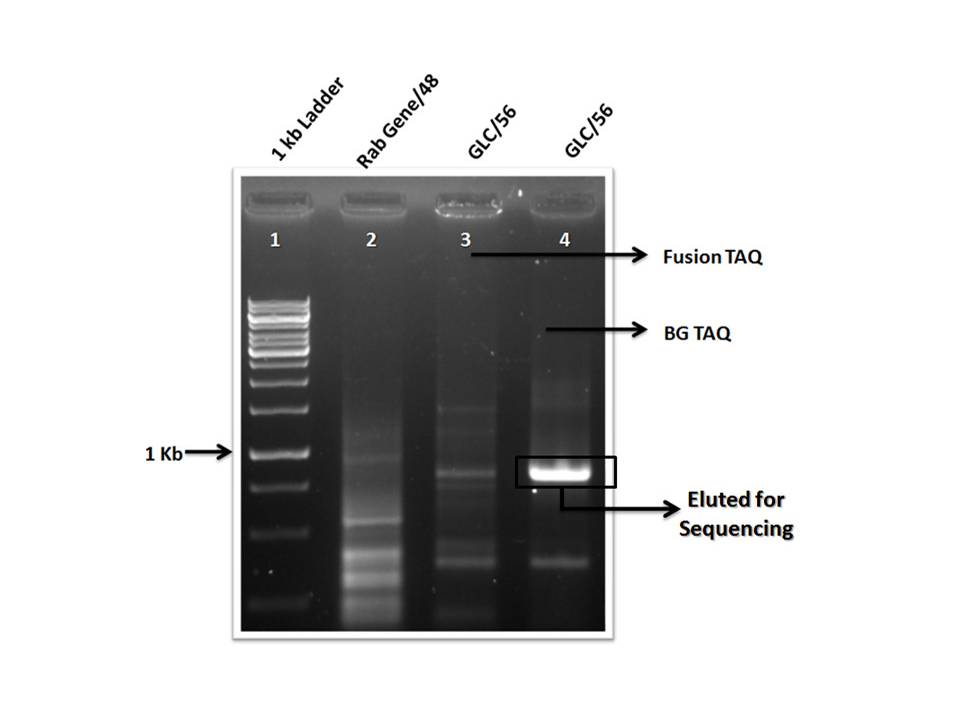


Fig. S1. Gel chromatogram depicting amplification of 1.0 kb DNA segment of β-1,3-glucanases on agarose gel


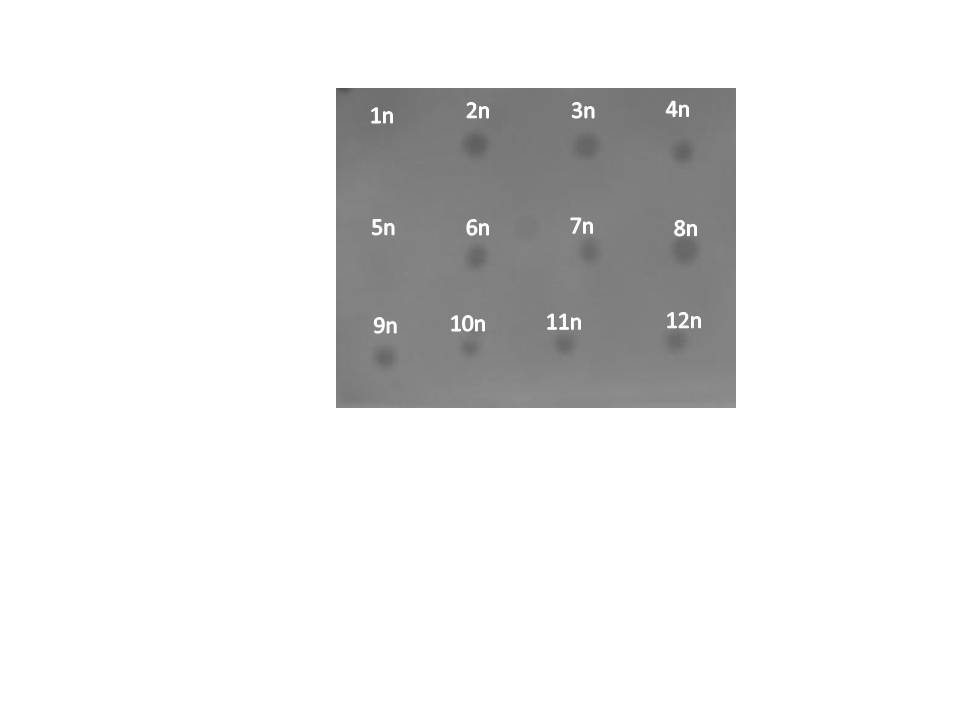


Fig. S2. RNA dot blot assay from PGPR inoculated paddy leaves in presence of pathogen. 1n spot is control, 2n spot is control + *B. pumilus,*3n spot is control *+ P. pseudoalcaligenes* and 4n spot is control + *B. pumilus+ P. pseudo alcaligenes* in absence of pathogen. 5n spot is control*,* 6n spot is control + *B. pumilus,*7n spot is control *+ P. pseudoalcaligenes* and 8n spot is control + *B. pumilus+ P. pseudoalcaligenes* in presence of pathogen. 9-12 spot eukaryotic translation factor eTEF1α as an internal standard.


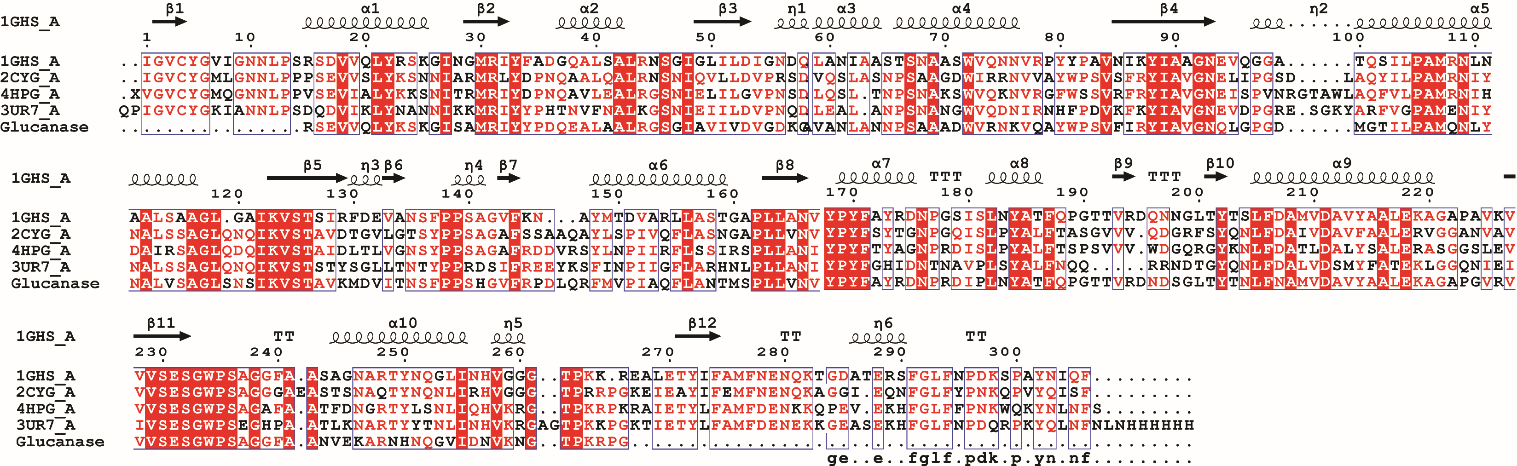


Fig. S3. Sequence-structure alignment of glucanase from *Oryza sativa* indica group with its structural homologs obtained after BLASTp search against PDB. The conserved residues are highlighted in red background with white color font. The secondary structures were assigned from the crystal structure of beta-glucan hydrolase from *Hordeum vulgare* (PDB ID: 1GHS, A chain)

**
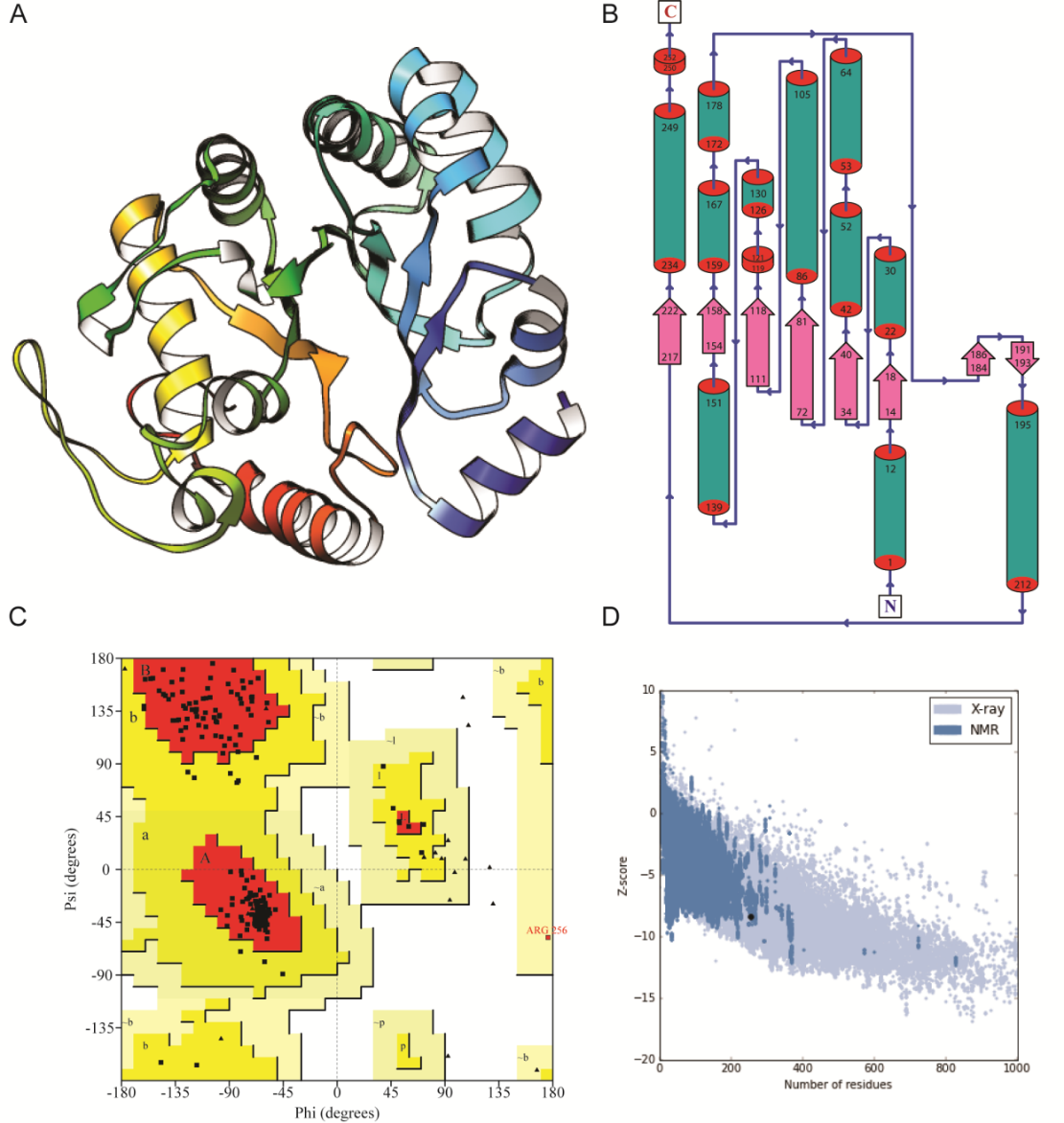
**

Fig. S4. Predicted 3-dimenisonal protein model of glucanase from rice. (A) Overall architecture of the modelled Glucanase. (B) Topology of the modelled glucanase from rice. (C) Ramachandran plot of the modelled glucanase evaluated using Procheck displaying none of the residues in the disallowed region indicating the high quality of the proposed model. (D) ProSA-web analysis displaying the z-score of the modelled glucanase structure.

**
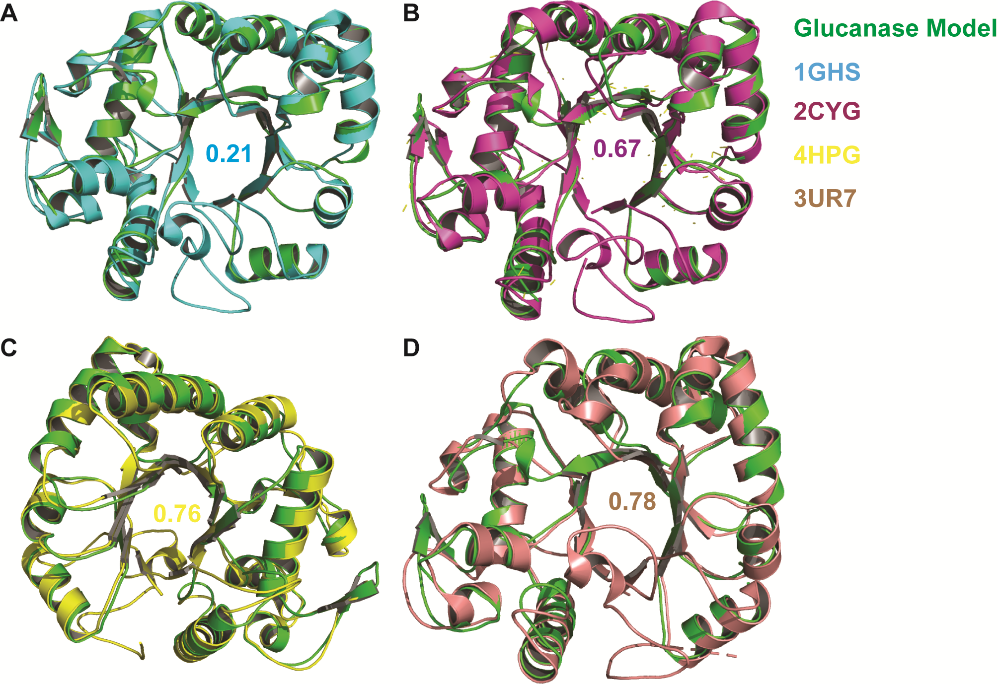
**

Fig. S5. Structural supposition of modelled Glucanase with the structural homologs displaying the overall Cα-RMSD (1GHS: Beta-glucan endohydrolase of *Hordeum vulgare*; 2CYG: endo-beta-1,3-glucanase of *Musa acuminata*; 4HPG: beta-1,3-glucanase of *Hevea brasiliensis*; 3UR7: endo-1,3-beta-glucanase of *Solanum tuberosum*).

**
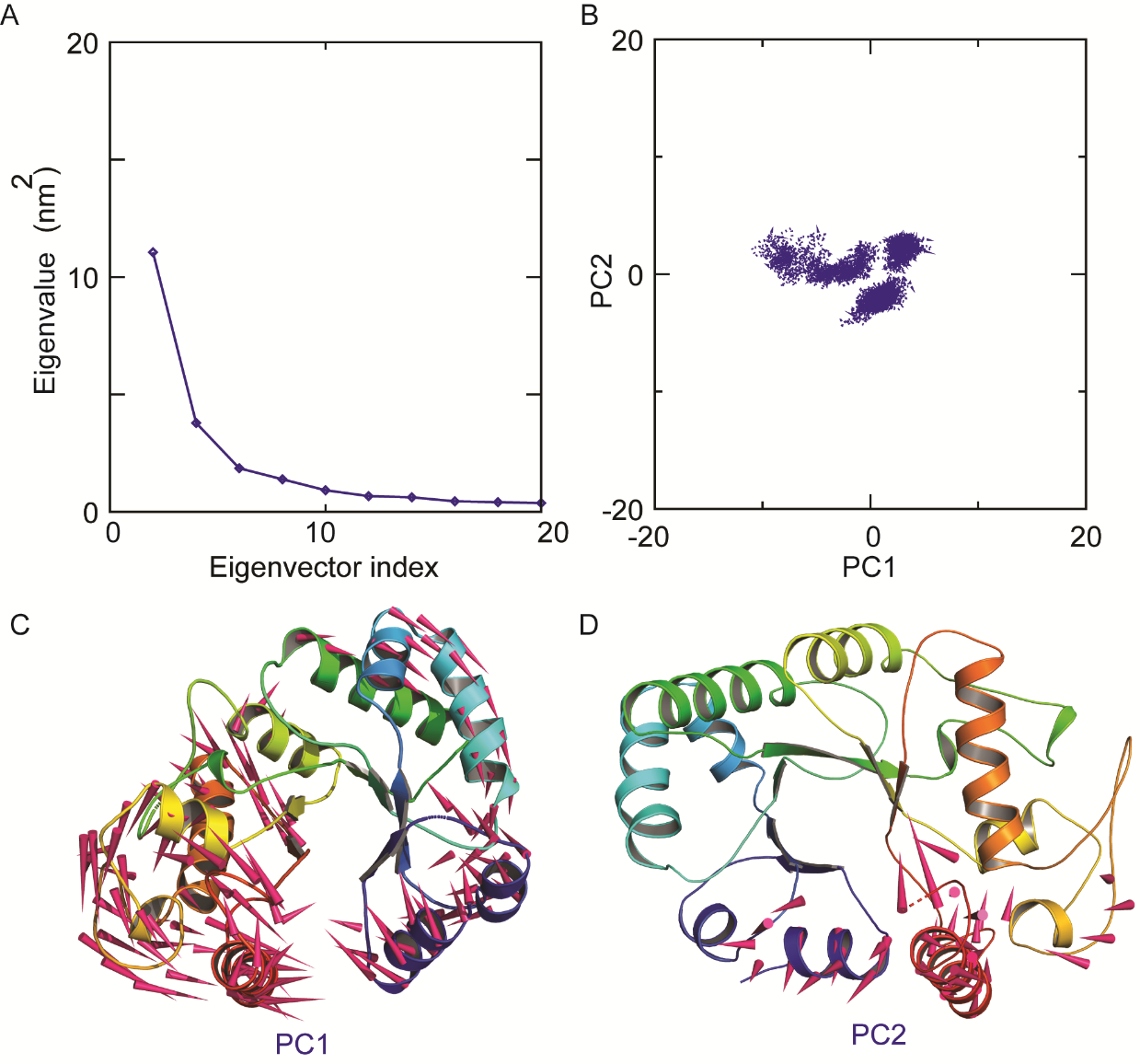
**

Fig. S6. Principal component analysis displaying prominent motions occupied by the first two principal components. (**A**) Eigenvalues of the first two eigenvectors. (**B**) Projection of the top two PCs in the phase space. (**C**) Porcupine plot displaying the global motion of PC1. (**D**) Porcupine plot displaying the global motion of PC2.
